# Supplementary material for: Effects of a Single Application of ScenarTM, a Low-Frequency Modulated Electric Current Therapy, for Pain Relief in Patients with Low Back and Neck Pain: A Randomized Single Blinded Trial
Source: J Clin Med. 2021 Nov 26;10(23):5570. doi: 10.3390/jcm10235570 (PMC8658569; doi:10.3390/jcm10235570)
Supplement: Supplementary file 1 [file jcm-10-05570-s001.zip › jcm-1443625 supplementary 1.pdf]

Table S1. CONSORT 2010 checklist of information to include when reporting a randomized trial*

Section and Topic	Item No	Checklist item	Reported on page No
**Title and abstract**			
	1a	The identification as a randomized trial in the title.	1
	1b	Structured summary of the trial design, methods, results and conclusions (for specific guidance see CONSORT for abstracts).	1
**Introduction**			
Background and objectives	2a	The scientific background and explanation of rationale.	1
	2b	The specific objectives or hypotheses.	1
**Methods**			
Trial design	3a	The description of the trial design (such as parallel and factorial) including the allocation ratio.	2 and 3
	3b	The important changes to the methods after the trial commencement (such as eligibility criteria), with the reasons.	NA
Participants	4a	The eligibility criteria for the participants.	2
	4b	The settings and locations where the data were collected.	4
Interventions	5	The interventions for each group with sufficient details to allow replication, including how and when they were actually administered.	3
Outcomes	6a	The completely defined prespecified primary and secondary outcome measures, including how and when they were assessed.	4
	6b	Any changes to the trial outcomes after the trial commenced, with the reasons	NA
Sample size	7a	How the sample size was determined.	4
	7b	When applicable, the explanation of any interim analyses and stopping guidelines.	NA
Randomization:			
Sequence generation	8a	The method used to generate the random allocation sequence.	2 and 3
	8b	The type of randomization and details of any restriction (such as blocking and block size).	2 and 3
Allocation concealment mechanism	9	The mechanism used to implement the random allocation sequence (such as sequentially numbered containers), describing any steps taken to conceal the sequence until the interventions were assigned.	2 and 3
Implementation	10	Who generated the random allocation sequence, who enrolled the participants, and who assigned the participants to the interventions.	2 and 3

Blinding	11a	If conducted, who was blinded after the assignment to the interventions (for example, participants, care providers and those assessing outcomes) and how.	3
	11b	If relevant, a description of the similarity of interventions.	3
Statistical methods	12a	The statistical methods used to compare the groups for primary and secondary outcomes.	4
	12b	The methods for additional analyses, such as subgroup analyses and adjusted analyses.	NA
**Results**			
Participant flow (a diagram is strongly recommended)	13a	For each group, the numbers of participants who were randomly assigned, received intended treatment and were analyzed for the primary outcome.	4
	13b	For each group, the losses and exclusions after the randomization, together with reasons.	NA
Recruitment	14a	The dates defining the periods of recruitment and the follow-up.	4
	14b	Why the trial ended or was stopped.	NA
Baseline data	15	A table showing the baseline demographic and clinical characteristics for each group.	4 and 5
Numbers analyzed	16	For each group, the number of participants (denominator) included in each analysis and whether the analysis was performed on the originally assigned groups.	4 and 5
Outcomes and estimation	17a	For each primary and secondary outcome, the results for each group, the estimated effect size and its precision (such as a 95% confidence interval).	4, 5 and 6
	17b	For binary outcomes, the presentation of both absolute and relative effect sizes is recommended.	NA
Ancillary analyses	18	Results of any other analyses performed, including the subgroup analyses and adjusted analyses, distinguishing the pre-specified from the exploratory analyses.	NA
Harms	19	All important harms or unintended effects in each group (for specific guidance see CONSORT for harms).	7
**Discussion**			
Limitations	20	The trial limitations, addressing the sources of potential bias, imprecision and, if relevant, the multiplicity of analyses.	8
Generalizability	21	The generalizability (external validity, applicability) of the trial findings.	
Interpretation	22	An interpretation consistent with the results, balancing the benefits and harms, and considering other relevant evidence.	7 and 8
**Other information**			
Registration	23	The registration number and name of the trial registry.	2
Protocol	24	Where the full trial protocol can be accessed, if available.	3
Funding	25	The sources of funding, other support (such as supply of drugs) and the role of funders.	9

*We strongly recommend reading this statement in conjunction with the CONSORT 2010 Explanation and Elaboration for important clarifications on all the items. If relevant, we also recommend reading the CONSORT extensions for cluster randomized trials, noninferiority and equivalence trials, nonpharmacological treatments, herbal interventions and pragmatic trials. Additional extensions are forthcoming: for those and for up to date references relevant to this checklist, see www.consort-statement.org.
